# Portal dosimetry correction method for validation of single isocenter VMAT plans for multiple brain metastases

**DOI:** 10.1002/acm2.13710

**Published:** 2022-08-12

**Authors:** Ronald J. Lalonde, M. Saiful Huq

**Affiliations:** ^1^ Department of Radiation Oncology UPMC Hillman Cancer Center Pittsburgh Pennsylvania USA

**Keywords:** brain metastases, portal dosimetry, treatment planning, VMAT

## Abstract

Portal dosimetry is one option for verification of volumetric‐modulated arc therapy (VMAT) planning for multiple brain metastases. However, due to the changing response of the portal imager with photon beam energy, the dose transmitted through closed multileaf collimator (MLC) leaves or narrow MLC gaps may be underestimated by the imager.

We present a simple method for correcting for these effects that may be implemented within the Eclipse treatment planning system. We recalculated the predicted portal dose with and without this correction for 20 multiple brain met VMAT plans. Before the correction, 3/20 composite plan fields passed our standard quality assurance (QA) criteria (54/80 individual fields); the average gamma passing rate for the composite plans was 76.9 ± 16.6%, and the average gamma value across the composite plans was 0.67 ± 0.23. After correction, 20/20 composite plan fields passed the QA criteria (80/80 individual fields); the average gamma passing rate for composite plans was 99.2 ± 1.4%, the average gamma value across the composite plans was 0.33 ± 0.90. A measure of plan complexity, the average leaf pair opening could be correlated to the gamma analysis results for the uncorrected plans but not for the corrected plans.

## 1 INTRODUCTION

Treatment of multiple brain metastases (mets) with a single isocenter volumetric‐modulated arc therapy (VMAT) plan has become an increasingly popular option for cranial stereotactic radiosurgery (SRS).^1^, ^2^, ^3^ During treatment of multiple brain mets, the linac jaws may be broadly open, even with the jaw tracking option turned on, delivering a substantial amount of radiation transmitted through closed multileaf collimator (MLC) leaves. Nonetheless, the single isocenter VMAT plans have been shown to compare favorably to other SRS delivery systems, including Gamma Knife and Cyberknife.^4^, ^5^

One challenge in treating patients with large numbers of mets in a single isocenter setup is performing robust plan quality assurance (QA) that will measure the dose delivered to all targets to the high resolution necessary for cranial SRS. Portal dosimetry^6^ is able to verify plan delivery for intensity‐modulated radiation therapy (IMRT), VMAT, and SRS plans^7^, ^8^ for both individual and composite fields. However, due to the high Z material in the phosphor layer and the relatively shallow depth of measurement, the portal imager is known to overrespond to low‐energy radiation.^9^, ^10^ Transmitted radiation filtered through the MLC, which has a higher overall energy spectrum, is then underestimated compared to radiation from an open field. This underestimation of the dose transmitted through the MLC may interfere with the accuracy of QA measurements for single isocenter VMAT fields for multiple mets.

In addition, much of the dose delivered in these plans is delivered through very narrow MLC leaf openings. In this case, a substantial portion of the dose is also delivered through partial transmission through the rounded leaf edge of the MLC. The difference between the light field position of the MLC and the radiation field delivered through the rounded leaf end of the MLC is referred to as the dosimetric leaf gap (DLG). Again, because the average energy of the beam delivered through a narrow MLC gap may be different from that of an open field, the DLG parameter used to correct for radiation dose delivered through a narrow gap may also be different as measured with the portal imager.

In this paper, we present a simple correction method that can be used to improve QA results for portal dosimetry for multiple brain met plans that can be accomplished quickly and easily within the Eclipse planning system (Varian Medical Systems, Palo Alto, CA, USA) itself. To the best of our knowledge, this is the first such study reported in the literature.

## 2 METHODS

We calculated and performed portal dosimetry verification plans for a series of 20 cranial SRS patients with multiple mets treated with a single isocenter (average 4.9 ± 2.8, range 2–14). All patient plans were calculated in the Eclipse planning system using the anisotropic analytical algorithm (AAA) v15.6 model for a 6XFFF beam on a TrueBeam STx linac, and all portal dosimetry plans were calculated using the PDIP v15.6 model. All plans were delivered on a Varian TrueBeam STx linac with an AS1000 electronic portal imaging device (EPID) imager.

In order to estimate the effect of the response of the portal imager on the MLC‐transmitted radiation dose, we repeated a sliding gap measurement^11^ to both a polystyrene slab phantom measuring dose with a PTW 31013 1.3 cc ion chamber at 5 cm depth and to the EPID. The sliding gaps ranged from 2 to 20 mm and were provided in a DICOM RT plan file provided by Varian Medical Systems. The transmission was measured for two closed leaf MLC patterns, one with the A leaf bank fully covering the field and the other with the B leaf bank covering the field, and an averaged value of MLC transmission was then used in dose calculation. For the portal dose images, the averaged EPID reading was recorded for a 5 mm × 5 mm area at the central axis of each field. If the sliding gap readings are then corrected for the relative amount of MLC‐transmitted dose, a plot of the readings versus gap size will have an intercept equal to the DLG.^11^

In clinical treatment planning, the values of the MLC transmission and DLG are determined by chamber measurements. However, in Eclipse version 15 and later, it is possible to create a separate AAA dose model with a modified value for MLC leaf transmission and DLG by adding a separate “add‐on” for the MLC and defining these values for the add‐on. This will then override the energy‐specific MLC transmission and DLG values defined for the linac in the RT administration task, but only for this one particular dose model. To correct the portal dose model for the response of the EPID, we made a copy of the clinical AAA model and created the add‐on MLC in the copy.

In the Eclipse planning system, the portal dose model will then use the MLC parameters of the plan calculated in AAA. For each patient plan, we then calculated the portal dose plan two times, once from the original patient plan and once from a copy of the patient plan with the modified MLC transmission and DLG values, calculated such that the MU's and MLC leaf positions were the same as in the clinical plan. The modified plans are referred to as “corrected plans” in this paper.

We then compared the results of the two portal dose predictions with the measured portal dose images and compared the following parameters: gamma pass rate (3% absolute dose/2 mm distance to agreement) for individual fields and composite images and the average gamma value of the composite images. Plans were considered to have passed validation if 95% of the pixels in the plan passed gamma criteria of agreement within 3%/2 mm, as suggested in Task Group‐218.^12^

One measure of the complexity of an IMRT/VMAT plan is the average leaf pair opening (ALPO). More highly modulated plans have a lower ALPO (<0.5 cm) and higher overall MUs to deliver equivalent doses. Since plans with a lower ALPO will have more dose delivered through either a closed MLC or a narrow MLC opening, we calculated per‐field and overall ALPO for all plans using a commercial software package (Radcalc, Lifeline Software) to see if this could be correlated with the results of gamma analysis for corrected and uncorrected plans.

## 3 RESULTS

For the “sliding gap measurements,” the MLC leaf transmission for the chamber/phantom setup was 0.0103 ± 0.0011 and the DLG was 0.579 ± 0.060 mm. For the portal imager, the MLC transmission and DLG values were 0.0069 ± 0.00025 and 0.152 ± 0.017 mm, respectively.

For the 20 patient plans, using the standard values of MLC transmission and DLG, only three out of 20 plans had composite images that passed our standard criteria of 95% of pixels passing gamma (3%/2 mm). For individual fields, 54/80 had greater than 95% of pixels passing gamma (3%/2 mm). The average gamma passing rate for the composite images was 76.9 ± 16.6%. The average of the averaged gamma values for the composite images was 0.67 ± 0.23.

**FIGURE 1:**
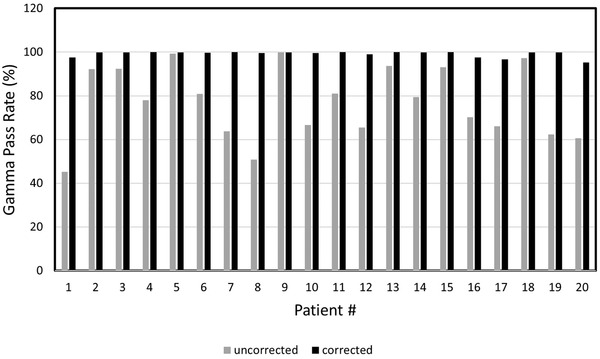
Gamma pass rates with and without correction applied for 20 patient composite portal dose images

With the adjusted values of MLC transmission and DLG, the composite images in all 20 plans passed our standard criteria, with an average gamma passing rate (3%/2 mm) for the composite images of 99.2 ± 1.4%. For individual fields, 80/80 fields had greater than 95% of pixels passing gamma (3%/2 mm). The average of the averaged gamma across the composite images was 0.33 ± 0.09. A comparison of the corrected and uncorrected gamma pass rates for all 20 plans is shown in Figure 1. The improvement in gamma pass rate varies between plans due to the relative amount of MLC‐transmitted dose in each plan.

**FIGURE 2:**
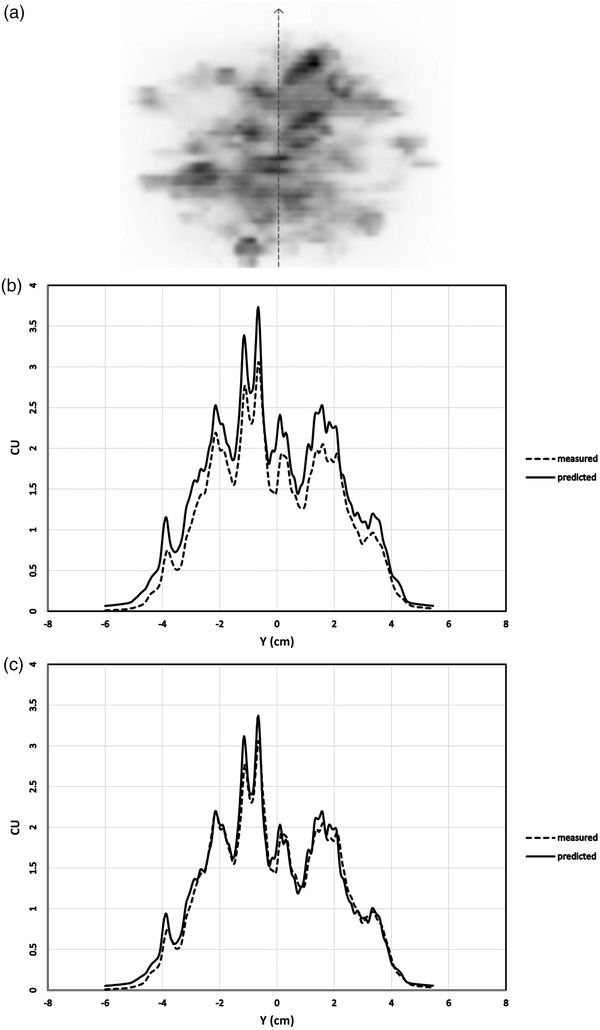
(a) Composite portal dose image from the multiple brain metastases volumetric‐modulated arc therapy (VMAT) plan. The dashed line shows the profile used for comparison of measured and predicted doses in (b) and (c). (b) Measured versus predicted portal dose across the *Y*‐axis of the composite portal dose distribution for the multiple metastases plan without correction for electronic portal imaging device (EPID)‐based multileaf collimator (MLC) transmission and dosimetric leaf gap (DLG). Varian portal dose images are displayed in calibrated units (CU). (c) Measured versus predicted portal dose across the *Y*‐axis of the composite portal dose distribution for multiple metastases plan corrected for EPID‐based MLC transmission and DLG

An example patient composite portal dose distribution is shown in Figure 2. Comparison profiles of measured and predicted portal doses for this composite image with and without the correction applied are shown in Figure 2.

**FIGURE 3:**
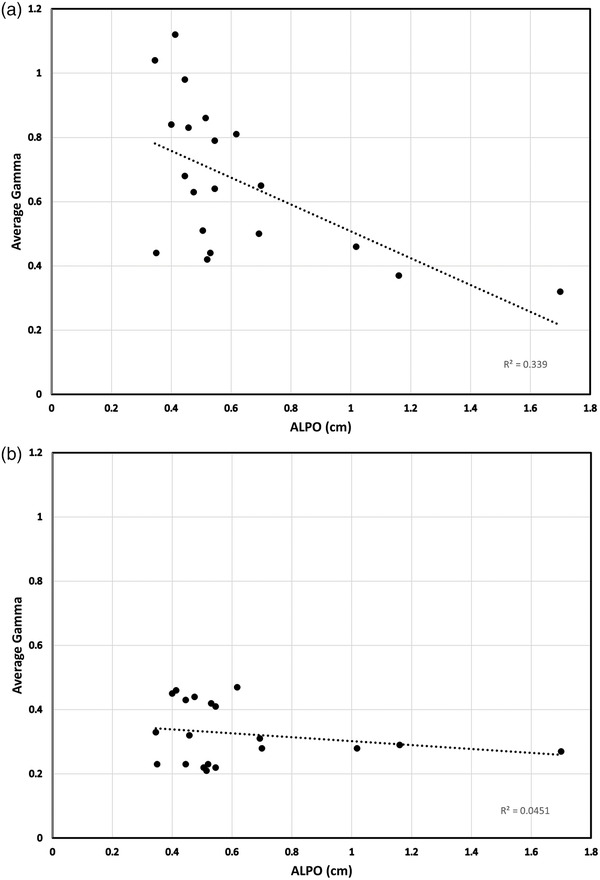
(a) Average gamma versus average leaf pair opening (ALPO) for composite portal dose analysis for all plans with no correction for electronic portal imaging device (EPID)‐based multileaf collimator (MLC) transmission and dosimetric leaf gap (DLG). (b) Average gamma versus ALPO for composite portal dose analysis for all plans corrected for EPID‐based MLC transmission and DLG

The ALPO for all patient plans ranged from 0.35 to 1.70 cm. There was a rough correlation (*R* ^2^ = 0.339) with the size of the ALPO for a plan and the averaged gamma value across the composite portal dose image for the uncorrected plans (Figure 3a). Plans with larger ALPO overall had smaller average gamma values, as expected. The correction method used here removes the systematic degradation of the gamma pass rate with MLC‐transmitted radiation, so there was a much smaller correlation with the corrected plans (*R* ^2^ = 0.0459, Figure 3b).

## 4 DISCUSSION

Our measurements of the ratio of the EPID to MLC‐transmitted radiation compared to a chamber measurement on the central axis (0.76) are similar to those previously reported by Vial et al.^9^ and Greer et al.^10^ (0.79 and 0.78, respectively).

Vial et al.^9^ previously described the variation in EPID response to MLC‐transmitted radiation, noting that it varied with off‐axis distance and field size. The mean energy of the beam increases with off‐axis distance due to the increased path through the MLC of the divergent beam. Increasing field sizes delivered an increased scatter dose from the MLC to the portal imager. They used a custom code to correct the portal images for each effect. However, the additional corrections for field divergence and field size effect are relatively small, <2% at off‐axis distances of <10 cm from the central axis (CAX). Most cranial VMAT fields will be well within this limit.

Their correction method, while more complete than the method described here, requires the user to export the predicted portal dose images, run a custom code, and then reimport them into the treatment planning system. The current method requires no coding and can be accomplished entirely within the existing planning software.

Similarly, Nicolini et al.^13^ describe an empirical method of recalculating EPID images including corrections for transmitted radiation such that the transformed EPID images can be compared to Eclipse dose calculations in water at dmax. Again, this method requires an additional software package and manipulation of portal images outside of the planning system.

The method described here could technically be applied to all plans. However, we have seen that the improvements in QA results to single target plans are not significant. This is because the main improvements are seen in areas with large amounts of dose transmitted through closed MLC leaves, as seen in multiple target plans.

It may be argued that some of the degradation in the QA results for these highly modulated plans might be due to error in MLC position or the incomplete modeling of the transmission through the rounded leaf of the MLC in the Varian dose model. These errors would have a larger effect on plans with narrower MLC openings. However, as shown in Figure 3b, the effect of any error in MLC leaf modeling is not enough to show a detectable correlation with ALPO or, as shown in Figure 1, to cause any of the corrected treatment plans to fail our QA acceptance criteria.

The method described here does require the user to maintain two copies of the AAA photon dose model, one with erroneous MLC transmission and DLG values that could, if applied to patient plans, lead to errors in dose delivery. Several steps have been taken to prevent the clinical use of this altered dose model. First, the machine name in the model has been changed to reflect that it is not for clinical use but for portal dose correction only. In addition, the absolute dose scaling factor in the altered AAA dose model has been changed by a factor of 1000; thus, any plan mistakenly calculated with the altered dose model would have MU values that far exceeded the deliverable MU limits of the machine.

## 5 CONCLUSION

VMAT plans for multiple brain mets include a large fraction of the dose delivered through closed MLC leaves or through a narrow gap between leaves, which will have a higher average energy than the open beam. The response of the EPID is known to vary with the energy of the beam. A method to correct the predicted portal dose model for these effects has been developed and shown to greatly improve the agreement between the predicted portal dose and measured QA plans. The method can be easily adopted using the existing treatment planning software.

## CONFLICT OF INTEREST

The authors declare they have no conflicts of interest.

## AUTHOR CONTRIBUTIONS

Ronald J. Lalonde developed the method described in this paper, performed all treatment plan calculations and predicted/measured dose comparisons, and drafted the manuscript. M. Saiful Huq reviewed the method and contributed valuable suggestions for the data analysis and formatting of the manuscript. Both authors gave final approval to the manuscript.
